# Editorial: Vaccine-induced innate immunity and its role in viral infections

**DOI:** 10.3389/fimmu.2024.1440061

**Published:** 2024-07-11

**Authors:** Mohammad Arif Rahman, Tesfaye Gelanew, Soumik Barman, Firzan Nainu

**Affiliations:** ^1^ Animal Models and Retroviral Vaccines Section, National Cancer Institute, National Institutes of Health (NIH), Bethesda, MD, United States; ^2^ Viral Diseases Research Division, Armauer Hansen Research Institute, Addis Ababa, Ethiopia; ^3^ Precision Vaccines Program, Boston Children’s Hospital, Boston, MA, United States; ^4^ Department of Pediatrics, Harvard Medical School, Boston, MA, United States; ^5^ Department of Pharmacy, Faculty of Pharmacy, Hasanuddin University, Makassar, Indonesia

**Keywords:** innate immunity, adaptive immunity, COVID19, T cells, antibody, neutralizing antibodies, vaccine, BCG

## Vaccine-induced innate and adaptive immunity in viral infections

This collection of articles compiles a range of contributions exploring the innate and adaptive immune responses elicited by both vaccination and infection. The role of adaptive immunity has been extensively studied for different vaccines and diseases, but less focus has been given to innate immunity. Studies have shown that epigenetic changes in the innate immune system might lead to long-lasting innate memory responses, also known as “trained immunity” (1, 2). However, the direct and indirect role of these “memory” innate immune responses on both specific and unrelated infections are not well studied.

The Bacille Calmette-Guérin (BCG) vaccine was initially developed to combat *Mycobacterium tuberculosis* (3, 4), and it has been widely used as an efficacious vaccine against childhood tuberculosis for the past 100 years (3–5) as well as other non-related infections (6–13). The major drawbacks of BCG are its waning immunity and lower efficacy against adult pulmonary tuberculosis infection (3, 4). Unique BCG-induced innate immunity and its trained memory are probably independent of adaptive immunity (14). Since pigs share many anatomical and physiological similarities with humans, Jensen et al. used a pig model to study the innate immunological responses generated by the BCG vaccine. The pigs showed no skin reactions, such as abscesses, ulcers, or scars, like what has been observed in humans. Live BCG bacteria were recovered from the draining lymph nodes of some of the animals up to 20 weeks after vaccination, suggesting that the bacteria contained in the vaccine were viable in pigs and that pigs cannot eliminate them promptly. Furthermore, they observed mycobacterial antigen-specific IFN-γ responses and post-vaccination changes in the expression of antiviral genes RIG-I and CSF1. However, these gene signatures disappeared after correction for multiple testing. Upon influenza challenge, the acute phase protein response was significantly reduced in BCG-vaccinated animals, suggesting that BCG induced trained immunity was able to control virus to some extent. However, further research is required to understand whether BCG-vaccinated pigs can be used to study the cross-reactive “memory-like” innate immunological responses that have been seen in humans.

The larger implications of BCG-induced innate immunity have been extensively studied recently due to the COVID-19 pandemic. In countries with high rates of BCG vaccination such as Ethiopia, Bangladesh, India, the Philippines, Thailand, and Nepal, epidemiological data have indicated that the mortality rate of COVID-19 infection was relatively low (15–17). However, the heterologous effects of COVID-19 vaccines have not been investigated in children. Noe et al. studied the specific and non-specific responses generated by BNT162b2 COVID-19 vaccination in children. They stimulated whole blood with a vast array of antigens and observed substantial reduction of IFN-γ, and MCP-1 responses, along with IL-6, IL-15, and IL-17 responses against *S. aureus*, *E. coli*, *L*. *monocytogenes*, BCG vaccine, *H. influenzae*, hepatitis B antigen, poly (I:C), and R848. Furthermore, immunized children also showed vaccine-specific responses. Taken together, the study suggested a heterologous response generated by the BNT162b2 COVID-19 vaccine, which might help to protect against unrelated infections.

Despite studies increasingly showing that vaccines and infections induce robust adaptive immune responses against the SARS-CoV-2 (18), there is a paucity of understanding in the nature and mechanisms by which adaptive immune responses are enhanced after primary and/or secondary vaccination and infection. Sheetikov et al. focused on healthy volunteers immunized with the SARS-CoV-2 spike protein incorporated Ad5-nCoV adenoviral vaccine to study the adaptive T cell responses. Due to the lack of studies on antigen-specific T cell repertoire generated by the Ad5-vector-based COVID-19 vaccine, the group conducted a longitudinal phase 3 clinical trial in Russia, monitoring SARS-CoV-2 specific T cell clonotype six months post-vaccination. *In vitro* stimulation and subsequent T cell sequencing analysis showed that people vaccinated with Ad5-nCoV adenoviral vaccine have a more diverse CD4^+^ T cell repertoire compared to CD8^+^ T cell. Additionally, they observed immunodominant epitope “NYNYLYRLF” specific CD8^+^ T cells, which were the predominant CD8^+^ T cell clonotypes in these vaccinated volunteers. This study suggested that the Ad5-nCoV vaccine generates strong and durable T cell responses, however, further study is needed to understand the protective role of these cells against SARS-CoV-2 infection.

To understand the humoral immune response against SARS-CoV-2 infection, Serwanga et al. studied a single dose of Janssen Ad26.COV2.S COVID-19 vaccine responses in a Ugandan cohort and investigated SARS-CoV-2 specific antibody responses against spike and nucleocapsid proteins. By day 14 post-priming with the vaccine, they observed anti-spike antibody response positivity rates were 98% and 86% for IgG and IgA, respectively and these robust antibody responses persisted throughout the 12 months observation period. On the other hand, the IgM positivity was suboptimal among the vaccinated individuals and became 0% by the end of the 12-month observation period. Furthermore, Li et al. analyzed neutralizing antibody levels following omicron COVID-19 infection in children from Beijing. Children who received inactivated SARS-CoV-2 vaccine prior to infection had higher levels of neutralizing antibodies compared to infected children with no vaccination history. Administering two doses of inactivated SARS-CoV-2 vaccine before infection significantly enhanced humoral immunity in pediatric populations, producing elevated neutralizing antibodies for up to three months post-infection. Zhao et al. showed that bivalent booster vaccination and past infections have enhanced neutralization against the XBB1.5 strain, however individuals with comorbidities exhibited reduced responses. Liao et al. investigated the effectiveness of booster immunization in China against Omicron BA.2 susceptibility, infectiousness, and transmission. Inactivated COVID-19 booster vaccination showed moderate protection against Omicron BA.2 infection and a low level of protection against transmission. Taken together, these studies highlight the necessity for ongoing vaccine updates to address emerging SARS-CoV-2 variants.

We hope readers will find this body of work to be a valuable reference for the latest advancements in understanding how vaccines and infections shape immune responses and their potential for cross-reactivity with related and unrelated pathogens, which ultimately will better prepare us for future epidemics and pandemics.

## Author contributions

MAR: Writing – original draft, Writing – review & editing. TG: Writing – review & editing. SB: Writing – review & editing. FN: Writing – review & editing.
